# Treatment of metastatic urachal adenocarcinoma in a young woman: a case report

**DOI:** 10.1186/1757-1626-2-9145

**Published:** 2009-12-04

**Authors:** Elmehdi Tazi, Issam Lalya, Mohammed Fadl Tazi, Youness Ahallal, Hind M'Rabti, Hassan Errihani

**Affiliations:** 1 Department of Medical Oncology, National Institute of Oncology, Rabat, 47000, Morocco; 2 Departement of Urology, Teaching Hospital Hassan Ii, Fez, Morocco

## Abstract

A 30-year-old woman with a history of smoking presented with abdominal pain and haematuria. On physical examination, she had a palpable pelvic mass. Imaging revealed a large pelvic mass located on the dome of the bladder, extending from the urachus, with pulmonary metastases. After open biopsy, urachal adenocarcinoma was histologically confirmed. The patient received six cycles of palliative chemotherapy combination 5 fluorouracil and irinotecan with complete response on the pelvic mass and partial response estimated to more than 80% on pulmonary metastasis.

## Introduction

**Figure 1:**
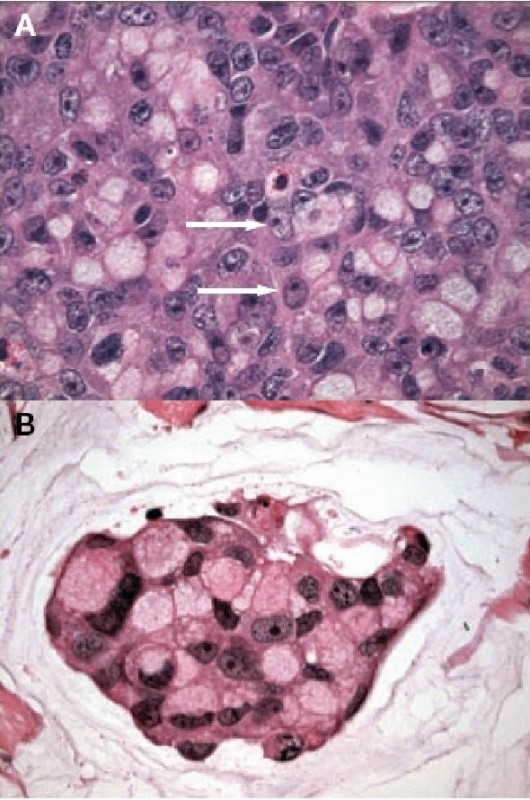
**Histological examination of the tumor**. **(A) **Sheets of poorly differentiated 'signet-ring' cells (arrows). **(B) **Nests of neoplastic cells floating in pools of extracellular mucin.

Primary adenocarcinomas of the bladder and urachus are extremely rare, accounting for 0.5-2.0% of all bladder malignancies. Although single-institution series have suggested that urachal adenocarcinomas account for 25-30% of primary adenocarcinomas of the bladder, a recent population-based analysis indicated that urachal adenocarcinomas represent approximately 10% of this group [1]. The urachus is a musculofibrous band that extends from the dome of the bladder to the umbilicus. During fetal development, the urachus develops into the median umbilical ligament that stretches from the umbilicus to the bladder. Urachal carcinoma stems from the epithelium of the remnant of this structure and adenocarcinoma accounts for 90% of all cases. Pathological examination of specimens of urachal carcinoma can reveal fragments of adenocarcinoma that float within 'pools' or 'lakes' of mucin (Figure 1). Patients with urachal carcinoma most commonly present with dysuria, hematuria, abdominal pain, or umbilical discharge [2]. The diagnostic evaluation for urachal carcinoma should include a careful history and physical examination. An urinalysis with cytology may be helpful. CT or MRI scans of the abdomen and pelvis can provide information on local extent of the disease, pelvic lymph-node involvement, and liver metastases. A cystoscopy is necessary to evaluate whether the carcinoma has penetrated the urothelium of the bladder and a transurethral biopsy should be performed if possible. To evaluate the presence of metastatic disease in the lungs or bone, chest X-rays and/or bone scans should be obtained. The MD Anderson Cancer Center has developed practical criteria for the diagnosis of urachal cancer and Sheldon *et al. *has proposed a staging schema for urachal carcinomas [3]. While early stage tumors are localized within the urachal mucosa, advancedstage lesions can extend into local structures such as the bladder, abdominal wall or peritoneum, and metastases to regional lymph nodes or distant sites [2]. A staging schema proposed by investigators at the Mayo Clinic highly correlated to the staging system proposed by Sheldon *et al. *and was advocated for its simplicity, however, owing to the limited numbers of cases, no staging system has been validated. [4].

## Case peresentation

**Figure 2:**
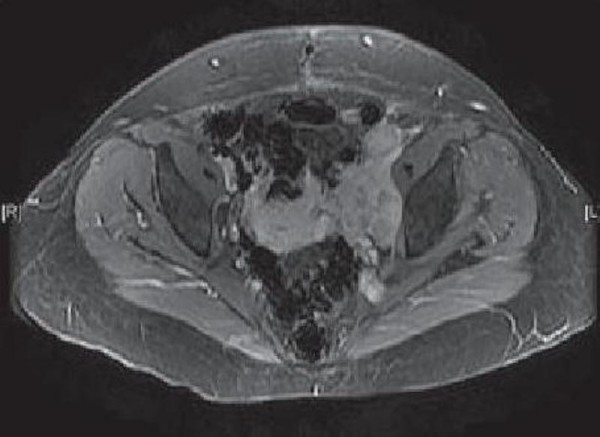
**MRI showing the extent of disease before therapy: Cross-sectional view of lobulated left pelvic mass measuring 7.8 × 6.6 cm**.

**Figure 3:**
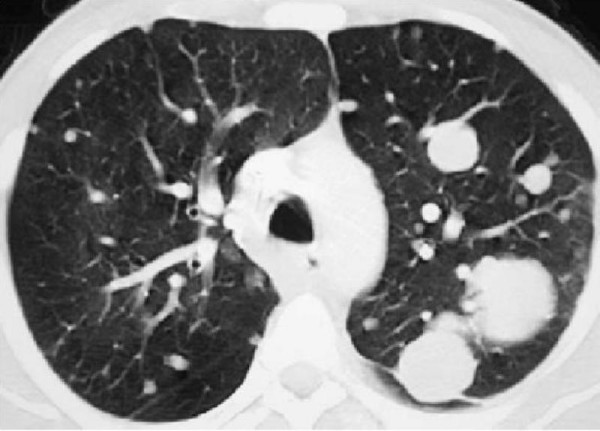
**CT scan view of pulmonary metastasis**.

**Figure 4:**
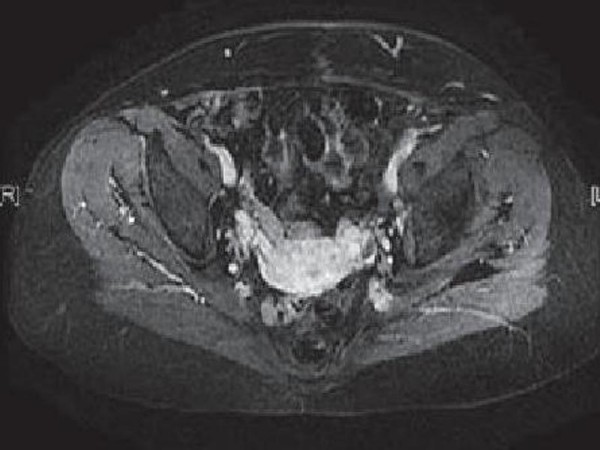
**MRI showing the extent of disease after therapy: Resolution of the left pelvic mass after chemotherapy**.

A 30-year-old Moroccan woman presented to her primary care physician having experienced abdominal pain, dysuria, and episodic gross hematuria for 1 month. Her medical history was unexceptional except for a 10-pack-year smoking history. Physical examination revealed a palpable suprapubic pelvic mass. The rest of her examination including pelvic and complete lymph-node examinations was unremarkable. Initial laboratory tests were all normal. An MRI of the abdomen and pelvis obtained 3 months after surgery revealed a lobulated left pelvic mass measuring 7.8 × 6.6 cm in the area of the prior pelvic-sidewall mass (Figure 2). No obvious sites of distant metastases were noted. A chest CT scan revealed a multiple pulmonary metastasis (Figure 3) and bone scan were negative. The patient underwent a cystoscopy, which revealed an indentation at the dome of the bladder and normal mucosa, biopsy was performed. The histological examination showed a poorly differentiated carcinoma to areas with poorly differentiated 'signet-ring' cells (Figure 1A). In addition, the tumor had a mucinous appearance with nests of neoplastic cells floating in pools of extracellular mucin (Figure 1B). Immunohistochemical staining revealed that these pleomorphic cells were positive for cytokeratin 20 but negative for calretinin consistent with metastatic carcinoma. A diagnosis of urachal carcinoma with locoregional extension to the left pelvic wall was made. The patient began chemotherapy 2 weeks after the MRI with irinotecan at 240 mg/m2/day 1, bolus 5-fluorouracil (5-FU) at 350 mg/m2, and leucovorin at 25 mg/m2/day 1 to day 5, delivered every 3 weeks. Owing to significant diarrhea, the chemotherapy dose was reduced by 25% after the first cycle and the patient tolerated this dose for the subsequent cycles. MRI of the abdomen and pelvis performed after 6 cycles of chemotherapy revealed no evidence of disease (Figure 4). At the last follow-up, no new sites of metastatic disease were noted on imaging scans.

## Discussion

Primary treatment of potentially localized disease includes wide local excision of the urachus, umbilicus, and surrounding soft tissue combined with partial or radical cystectomy and bilateral pelvic lymphadenectomy [4,5]. Although radical cystectomy has historically been advocated, several studies have demonstrated long-term survival with extended partial cystectomy including *en bloc *removal of the umbilicus, urachal tumor mass, the entire urachal ligament, and bladder dome [3,4,6]. Despite primary treatment, a retrospective analysis of 66 patients with primary urachal carcinoma treated at the Mayo Clinic revealed a 5-year overall survival rate of only 49% [4,6,7]. Risk factors for recurrence include lack of *en bloc *resection of the umbilicus, peritoneal involvement, and positive nodes and/or margins [4,6,8]. Owing to lack of early symptoms, the cancer usually presents at an advanced stage. In a population-based study including 62 individuals with a primary diagnosis of urachal tumor, only one patient had cancer localized to the urachus [5]. Rates of local recurrence are high, with disease usually reoccurring in the pelvis or bladder within 2 years of surgery [2,6]. Chemotherapy and radiation for urachal adenocarcinoma have resulted in minimal responses with no definitive improvement in survival. The rarity of the cancer has prevented accrual to controlled clinical trials. Case reports describe results with various 5-FU and/or cisplatin-based regimens [9-12]. Despite measurable responses to treatment, tumors often recur and the majority of patients die within 2 years of diagnosis. Siefker-Radtke *et al*. Reported the results of a retrospective review of 42 patients treated at the MD Anderson Cancer Center. Among the 26 patients who developed metastases, only 4 had significant responses to chemotherapy, and of the 9 patients who received chemotherapy with 5-FU or cisplatin-containing regimens three responded [6]. A phase II trial with 5-FU, leucovorin, cisplatin, and gemcitabine for adenocarcinomas of the urachus is ongoing at the MD Anderson Cancer Center [3]. Irinotecan is a topoisomerase I inhibitor that disrupts cell division by interfering with DNA replication. Irinotecan has demonstrated preclinical activity in adenocarcinomas from a variety of tumor types including gastric, colorectal, pancreatic, lung and breast carcinomas [13]. Currently, irinotecan in combination with 5-FU/leucovorin with or without bevacizumab is indicated as first-line therapy for metastatic colorectal cancer [14]. Irinotecan has also demonstrated efficacy for metastatic gastric cancer in combination with 5-FU/leucovorin or cisplatin [15,16]. Urachal adenocarcinomas are often histologically similar to adenocarcinomas at other sites of origin, including those in then gastrointestinal tract such as the colon or stomach. The histology from this patient demonstrated the typical 'signet-ring' pattern that is also typical of gastric cancer. There is an additional case study in the literature from Japan that describes a patient with metastatic urachal carcinoma and a history of considerable chemotherapy whose lung lesions had a marked response to irinotecan.

## Conclusion

Currently, there is no standard chemotherapy regimen for the treatment of metastatic urachal adenocarcinoma. The demonstration that more recently developed agents have efficacy in adenocarcinomas from other tumor types should inspire treatment options for this difficult disease. In addition, agents that demonstrate efficacy in metastatic disease should be pursued in trials designed to clarify the role of neoadjuvant or adjuvant chemotherapy in urachal adenocarcinoma.

## Consent

Written informed consent was obtained from the patient for publication of this case report and accompanying images. A copy of the written consent is available for review by the Editor-in-Chief of this journal.

## Competing interests

The authors declare that they have no competing interests.

## Authors' contributions

ET analyzed and interpreted the patient data regarding the retroperitoneal disease. IL have made contributions to conception and design, and acquisition of data. YA and MT have been involved in drafting the manuscript and revising it critically for important intellectual content. HM and HE have given final approval of the version to be published. All authors read and approved the final manuscript.
